# New Ruthenium Nitrosyl Complexes Combining Potentially Photoactive Nitrosyl Group with the Magnetic Nitroxide Radicals as Ligands

**DOI:** 10.3390/ijms241713371

**Published:** 2023-08-29

**Authors:** Gennadiy A. Kostin, Ruslan Kozlov, Artem Bogomyakov, Svyatoslav Tolstikov, Dmitriy Sheven, Sergey Korenev

**Affiliations:** 1 Nikolaev Institute of Inorganic Chemistry Siberian Branch of Russian Academy of Science, Lavrentieva, 3, 630090 Novosibirsk, Russia; 2 Department of Natural Sciences, Novosibirsk State University, Pirogova, 2, 630090 Novosibirsk, Russia; 3 International Tomography Center Siberian Branch of Russian Academy of Science, Institutskaya, 3a, 630090 Novosibirsk, Russia

**Keywords:** ruthenium, nitrosyl, nitroxide, magnetic measurements, MS spectroscopy

## Abstract

Two ruthenium nitrosyl complexes of Na[RuNOCl_4_L] with nitronyl nitroxide radicals coordinated to ruthenium with N-donor pyridine rings were prepared and described. The crystal structure of both complexes is 1D or 2D polymeric, due to the additional coordination of sodium cation by bridging the chloride ligands or oxygen atoms of nitroxides. Partially, the oligomeric forms remain in the solutions of the complexes in acetonitrile. The magnetic measurements in the solid state demonstrate the presence of antiferromagnetic interactions through the exchange channels, with the distance between paramagnetic centers equal to 3.1–3.9 Å. The electrochemical behavior of the prepared complexes was investigated in acetonitrile solutions.

## 1. Introduction

Magnetic materials based on nitroxide radicals of different types have been of great interest for the last several decades. The magnetic exchange between nitroxide moieties and other paramagnetic centers can result in ferromagnetics [1], spin-crossover compounds [2], “breathing crystals” with the “single-crystal-to-single-crystal” transition [3], solvent sensible magnetics [2,4], etc. Usually, the combination of organic radicals with paramagnetic metal ions is explored to achieve special magnetic properties. Still, diamagnetic cations can be used to modulate the interactions between radical centers in the solid state [5,6].

The idea of optically switched magnetic materials was realized for the first time for the example of mixed cobalt iron cyanide (Prussian blue type) [7]. Since that time, a large number of photo-switchable magnetics have been prepared on the basis of cyanide complexes [8,9], complexes with switching spin crossover [10], and organic photo-switchable ligands [11,12,13]. Photo-switching can be realized directly in the magnetic center by changing the Jahn–Teller effect or the spin state of the paramagnetic ion [10,14,15]. Another approach is based mainly on the switching of structure by changing the distance between paramagnetic centers [12,13].

Ruthenium nitrosyl complexes are one of the most interesting examples of a photo-switching group, since the Ru-NO moiety can exist in three different isomeric forms [16,17,18]. The most stable is the ground state (GS), in which the Ru-NO (N-coordinated NO) group can be transferred by light irradiation with a specific wavelength to metastable MS1 (Ru-ON, O coordinated) or MS2 (Ru < NO, η2 coordinated). All three isomers possess different physical and chemical characteristics, in particular the well-defined photochromism. Thus, it can be considered as a three-position switch in contrast to most of the switching fragments.

In our previous work, we have shown the principal possibility of combining nitronyl nitroxide radicals (L) with ruthenium nitrosyl, and reported the first structure of Na[RuNOCl_4_L] [19]. The present paper is devoted to the development of our approach and the investigation of some of the properties of prepared complexes. For the first time, we describe the magnetic properties of such complexes and investigate their electrochemical behavior.

## 2. Results and Discussion

### 2.1. Structure and Synthesis

Earlier, we investigated the reaction of Na_2_[RuNOCl_5_] with N-donor heterocycles to prepare the first example of [RuNOCl_4_L]^−^ complexes with a nitronyl-nitroxide radical [19]. In the present paper, we examine the approach to the synthesis of complexes with similar ligands. Since the nitroxides themselves are relatively weak ligands, that could be crucial for inert ruthenium complexes, we exploited nitroxides with an additional N-donor center. For both the investigated ligands, the only complex with a *cis*-position of NO and L is formed, according to single-crystal X-ray diffraction. The vibration spectra of both complexes contain the only absorption band corresponding to ν(NO) vibrations (1872 and 1868 cm^−1^) that could be assigned to the *trans*-NO-Ru-Cl coordinate. The vibrations of the N-O fragment in a nitronyl nitroxide ligand are usually located at 1370–1400 cm^−1^ [20]; thus, the strong band in the spectra of both compounds at 1372–1373 cm^−1^ could be assigned to the nitroxide NO stretching vibrations.

Generally, the substitution of one chloride ligand to N-heterocycle (L) in [RuNOCl_5_]^2−^ can result in the formation of a *trans*(L,NO)- or *cis*(L,NO) isomer. Arion et al. reported that the reaction of [RuNOCl_5_]^2−^ with different amino acids (AA) results in only one isomer, namely *mer*(Cl)-*trans*(O, NO) [RuNOCl_3_(AA-H)] [21]. The reaction of [RuNOCl_5_]^2−^ with N-heterocycles in different alcohols with the molar ratio heterocycle Ru < 2 results in both *cis*- and *trans*-isomers of [RuNOCl_4_L]^−^; the longer reaction time seems to be favorable for *cis*-isomer formation [22]. The reaction of [RuNOCl_5_]^2−^ with the 5–10 molar excess of N-heterocycles in boiling DMF only results in *fac*-[RuNOCl_3_L_2_] [23]. The strong π-acceptor properties of the NO ligand result in the strong labialization of the trans-to-NO ligand due to the kinetic trans-effect [24]; thus, the *trans*-isomers seem to be the products of the kinetic control, while the formation of *cis*-isomers results from the thermodynamic control of the reaction.

Both new crystal structures (**2**)–(**3**), as well as the previously reported structure (**1**), are built from the anionic [RuNOCl_4_L]^−^ fragments, which are combined into 1D or 2D structures due to the additional interactions of the chloride ligands or oxygen atoms of nitroxide radicals with sodium cations. The structural parameters of the [RuNOCl_4_L]^−^ moiety in all three structures (Table 1) are close to typical for nitrosyl complexes [23,25,26]. The Ru-Cl distances vary in the range of 2.34–2.40 Å. The Ru-NO bond and the bond between nitrogen and oxygen atoms in the nitrosyl group are quite short, due to the dual nature of the NO ligand, which is both the σ-donor and π-acceptor. The RuNO fragment is close to linear in all the investigated compounds. That kind of geometry is typical of ruthenium nitrosyl complexes, whose electronic structure can be represented as Ru^2+^-NO^+^. In all structures, an interplane angle between the pyridine ring and nitroxide fragment sufficiently deviates from the coplanar, prohibiting the distribution of spin density on the pyridine ring.

**Figure 1:**
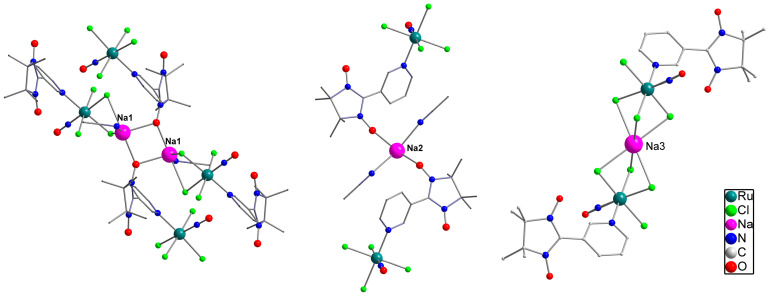
The different crystallographic positions of sodium cations in the crystal structure of (**2**). Hydrogen atoms are omitted for clarity.

Structure (**2**) crystallizes in the triclinic (P-1) group. The asymmetric unit consists of two ruthenium anions, two sodium cations, and one acetonitrile molecule. In the crystal structure (**2**), 2D layers are formed by the additional interactions between sodium cations and the oxygen atoms of nitroxide radicals or bridging chloride ligands. There are three independent positions of sodium atoms in the crystal structure (Figure 1). In the first position, Na1 is coordinated by three bridging chloride ligands with Na-Cl distances of 2.798(3)–2.800(3) Å, two oxygen atoms from two nitroxide radicals with Na-O distances being equal to 2.323(3)–2.415(3) Å, and an additional acetonitrile molecule with an Na-N distance equal to 2.450(2) Å. The coordination environment of Na1 can be represented as a distorted square pyramid with two chlorine atoms, an oxygen atom and a nitrogen atom at the base. The sodium cation in the second position is disordered in two positions with equal occupation (50%). The coordination environment of Na2 can be represented as a distorted square with a trans-arrangement of two coordinated oxygen atoms from the nitroxide radicals (2.233(2) and 2.356(2) Å) and two nitrogen atoms from the acetonitrile molecules (2.512(3) and 2.507(3) Å). The sodium cation Na3 is in the private position (0.5, 0.5, 0). The coordination environment of Na3 is a distorted octahedron from six chlorine atoms with distances of 2.681(3)–2.849(3) Å.

**Figure 2:**
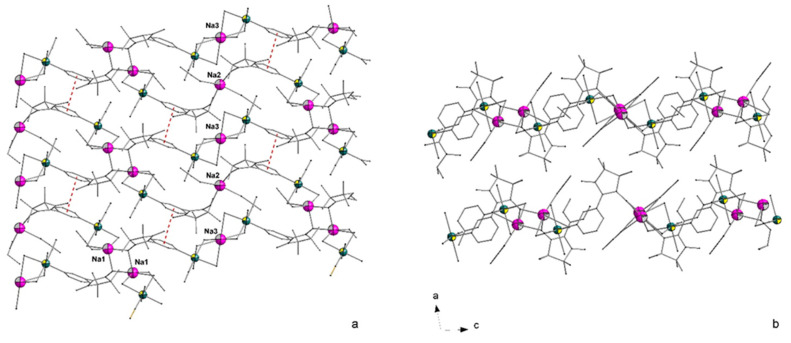
(**a**) 2D layer in the structure (**2**). Stacking interactions are marked with red dashes. (**b**) The view of structure (**2**) along the *b* axis. Hydrogen atoms are omitted for clarity.

In two-dimensional grids oriented perpendicular to the *a* axis, rows formed by Na1 fragments alternate with rows formed by Na3 and Na2 fragments (Figure 2). Additionally, the network is stabilized by -pi-stacking interactions between the pyridine rings of the ligands with distances between the ring planes of 3.786 Å. According to the CCDC database, bridging sodium cations are not rare for the crystal structures of chloride complex salts. Similar short Na…Cl contacts in the solid state were revealed for different complexes of transition metals, such as ruthenium [27,28], iridium [29], and rhenium [30]. The Na…O distances in the studied structures are also short enough to be considered as coordination bonds. Similar distances were observed earlier for the sodium complexes of crown ethers [31,32].

**Figure 3:**
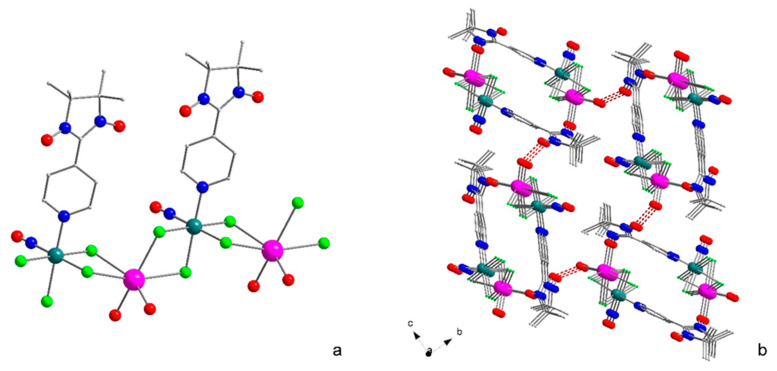
(**a**) Chain building block in structure (**3**). (**b**) View of the crystal structure (**3**) along the *a* axis. Hydrogen bonds are marked with red dashes. Hydrogen atoms are omitted for clarity.

Complex (**3**) crystallizes in the orthorhombic (P2_1_2_1_2_1_) space group. The asymmetric unit contains one ruthenium anion, one sodium cation, and a water molecule. The structure (**3**) is formed on the basis of the RuCl_4_-Na-RuCl_4_ chain motif, in which each sodium cation is bonded to the two neighboring ruthenium anions by the two bridging chloride ligands. The sodium coordination environment is a distorted octahedron of four chlorine atoms and two oxygen atoms from the nitroxide radical and water molecule. The Na-Cl bond lengths are in the range of 2.786(1)–3.004(1) Å and the Na-O distances are 2.281(3) and 2.299(3) Å. In the crystal lattice, these chains are oriented along the *a* axis and are linked by hydrogen bonds between the oxygen atom of the water molecule coordinated to sodium and one of the oxygen atoms of the nitroxide radical with an O…O distance of 2.72 Å (Figure 3). The presence of water in the crystal structure (**3**) is also confirmed by the IR spectra, where corresponding vibration bonds are located at 3580 cm^−1^ (ν(OH)) and 1616 cm^−1^ (δ(OH)).

### 2.2. Hirshfeld Surfaces Analysis

**Figure 4:**
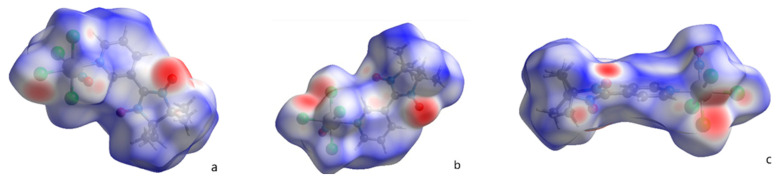
Hirshfeld surfaces for the anions in structure (**2**) (**a**,**b**)—two crystallographically independent units—and (**3**) (**c**).

A Hirshfeld surfaces analysis was performed for the anionic fragments [RuNOCl_4_L]^−^ in structures (**2**) and (**3**) to study the weak interactions in the complexes, such as hydrogen bond, π-π, molecular packing in crystal, and van der Waals interactions. The d_norm_ (Figure 4) and cool contact detachment provide a comprehensive relationship between the distances of any superficial point and the surrounding internal (d_i_) and exterior (d_e_) atoms along the van der Waals distance.

The mostly red zones in Figure 4 correspond to the earlier mentioned interactions between sodium cations and chlorine or oxygen atoms responsible for the polymeric nature of structures. Still, the analysis of 2D fingerprints allows additional weak interactions to be revealed, which include the ruthenium nitrosyl group or nitroxide groups. The contribution of certain atoms to the anion Hirshfeld surfaces in structures (**2**) and (**3**) is similar. The most significant contribution is made by hydrogen atoms (48% for both structures), chlorine atoms (29.8 for (**3**) and 29.9% for (**2**)), and oxygen atoms (9.7% for (**3**) and 13.3% for (**2**)) (Supplementary Materials Figure S3a,b).

**Figure 5:**
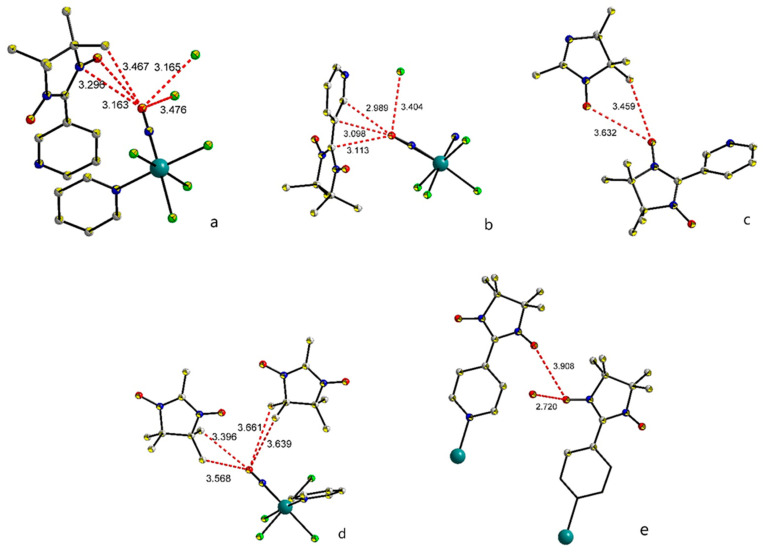
The shortest contacts of RuNO and nitroxide fragments in structure (**2**) (**a**–**c**) and (**3**) (**d**,**e**).

The relatively short contacts of ruthenium nitrosyl groups and nitroxide groups are shown in Figure 5. In structure (**2**), the distance between nitroxide oxygen atoms is equal to 3.632(2) Å and the additional interactions between nitroxide oxygen and the methyl groups of adjacent nitroxide are also revealed, with the distance O…C equal to 3.459(3) Å. In structure (**3**) the contacts between nitroxide fragments are longer with the distance O…O equal to 3.908(3) Å. The shortest contacts of nitroxide oxygens in structure (**3**) (2.720(3) Å) correspond to the weak hydrogen bonding with the water molecule coordinated to the sodium cation. The oxygen atoms of the nitrosyl group in both structures are surrounded by the nitroxide ligands of the neighboring [RuNOCl_4_L]^−^ fragments; in structure (**2**), the nitrosyl groups are also surrounded by the chlorine ligands of the neighboring fragments.

### 2.3. Mass-Spectrometry

**Figure 6:**
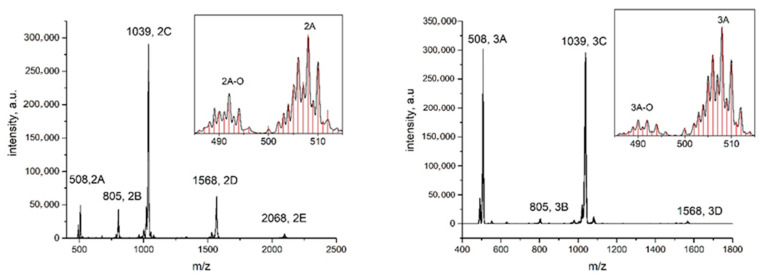
Mass spectra of the acetonitrile solutions of (**2**) and (**3**). The assignment of the main forms corresponds to Table 2. Experimental and calculated isotopic distribution patterns (red lines) corresponding to the [RuNOCl_4_L]^−^ form are shown on the insets.

The electrospray ionization mass-spectra (ESI-MS, negative ions—NI) of the investigated compounds in acetonitrile solutions reveals the presence of an [RuNOCl_4_L]^−^ anion for both complexes, and also the presence of oligomeric forms, corresponding to the parts of the molecular chains detected in the solid-state crystal structure. The forms were assigned based on the position of the most intensive line (Table 2) and verified by the characteristic patterns of isotopic distribution (Figure 6, Supplementary Materials, Figure S1a,b).

The presence of dimeric (**C**) and trimeric (**D**) forms was detected for both compounds. Moreover, for the complex with the ligand L^1^ (**2**), the signal of the dimeric form (**C**) was six times more intensive than for the monomeric anion (**A**). All the main signals (**A**), (**C**), (**D**) were accompanied by minor peaks with lower *m/z*, corresponding to the loss of one oxygen atom. The conversion of nitronyl nitroxide to an imino nitroxide radical is well-known and can be mediated by different factors (temperature, acidic media, the presence of reducing agents) [33,34,35]. Still, the EPR spectra of complex solutions in acetonitrile have only five specific lines corresponding to the nitronyl nitroxide ligand [19]; thus, the deoxygenated species (**A**)-O and (**C**)-O form in the process of ESI-MS.

### 2.4. Electrochemistry

**Figure 7:**
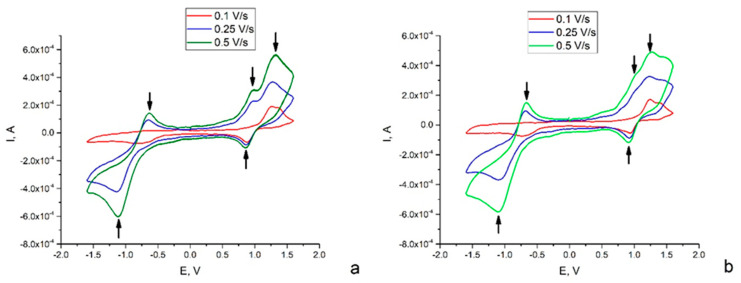
Cyclic voltammogram of a solution of 2.0 mM of (**2**) (**a**) and (**3**) (**b**) in acetonitrile containing 0.1 m TBAPF_6_ using a glassy carbon electrode with different scan rates. The arrows show the redox peaks mentioned in Table 3.

The redox properties of complexes (**2**) and (**3**) were investigated in acetonitrile solutions with different scan rates (0.1–0.5 V/s). Both complexes demonstrated irreversible ox-red behavior, and the corresponding cathodic and anodic potentials were strongly dependent on the scan rate. Complex (**2**) showed two irreversible reduction processes (Figure 7a). The potential of the first peak was weakly dependent on the scan rate (0.88–0.85 V), while the second one shifted from −0.739 to −1.120 V with an increase in the scan rate (0.1–0.5 V/s). Also, three oxidation peaks could be revealed. The first one (−0.651–0.633 V) occurred only at the higher scan rates (0.2–0.5 V/s), the potential of the second peak decreased (1.24–0.98 V) with an increase in the scan rate, and the third one had a potential equal to 1.32–1.35 V. Similar processes can be revealed for complex (**3**) (Figure 7b, Table 3).

Since the nitronyl nitroxide ligands are compounds with a one-electron shell lying between the corresponding hydroxylamine (strong reducing agent) and oxoammonium salt (strong oxidizer), the pure ligands usually demonstrate two quasi-reversible waves. The potentials of the oxidation process (nitroxide–oxoammonium salt) are in the range of 0.4–0.8 V and the electron acceptors shift the potentials to higher values [36,37,38,39,40]. The potentials of the reduction process (nitroxide–hydroxylamine) varies from −1.4 to −0.8 V and the electron acceptors also shift the potentials to higher values [36]. Thus, we can suppose that the cathodic peaks are related to the nitroxide transformations, as well as the anodic peaks, with potentials of 0.6 and 1–1.2 V. The irreversible oxidation process at potentials of 1.2–1.4 V can be assigned to the oxidation of the (RuNO)^3+^ group according to the literature data for ruthenium nitrosyls [25,41].

If the redox process is chemically and electrochemically reversible, the difference between the anodic and cathodic peaks, called peak-to-peak separation, is close to 59 mV (2.303RT/F at 25 °C). The redox processes associated with nitroxide ligands (−0.7 and 0.9–1.2 V) obviously do not fit that requirement. Moreover, the peak-to-peak separation shifts with an increase in the scan rate, which indicates electrochemical quasi-reversibility [42,43]. Earlier, it was shown that pure ligands demonstrate reversible redox transformations [36,37,38,39,40]. The most probable reason for the irreversibility in the investigated complexes is the presence of the chemical reaction conjugated to the redox process, e.g., the interaction of a reduced ligand with the Ru-NO fragment. Nevertheless, a rigorous confirmation of this hypothesis requires additional studies.

### 2.5. Magnetic Measurements

**Figure 8:**
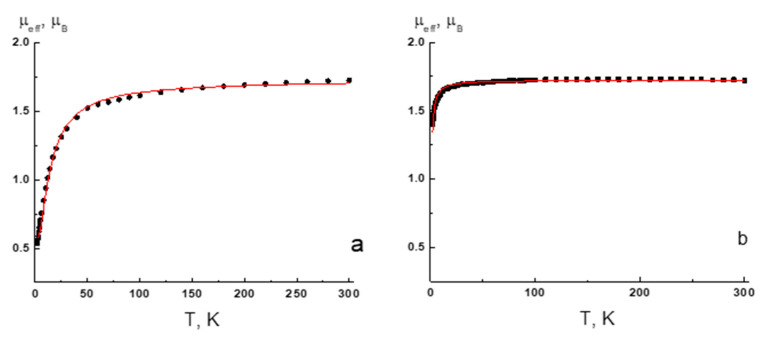
The µ_eff_(T) dependencies for complexes (**1**) (**a**) and (**3**) (**b**). Solid lines are theoretical curves.

The temperature dependencies of the effective magnetic moment (µ_eff_) for complexes (**1**) and (**3**) are shown in Figure 8. The µ_eff_ values at 300 K are very close to a theoretical spin-only value of 1.73 µ_B_ for the monoradical of the molecular mass, corresponding to formulas (**1**) and (**3**). For complex (**1**) the µ_eff_ changes slightly as the temperature is lowered, and then decreases to 0.54 µ_B_ at 2 K. The decreasing in µ_eff_ is indicative of antiferromagnetic coupling due to short contacts (NO....ON 3.121 Å, NO...CH3 3.169 Å) (Supplementary Materials Figure S2a) between the paramagnetic fragments of nitroxides in the solid (**1**). An analysis of the experimental µ_eff_(T) dependence with an alternating chain model (Spin Hamiltonian *H = –2J_1_·S_i–1_S_i_ – 2J_2_·S_i_S_i+1_*) results in equal J_1_ and J_2_ values, so the uniform chain model was used (*H = –2J·S_i_S_i+1_*). The best-fit values of the g-factor and exchange coupling parameter J are 2.00 ± 0.01 and −7.7 ± 0.1 cm^−1^. In the case of complex (**3**), the µ_eff_ decreases below 25 K to 1.40 µ_B_ at 2 K. According to the X-ray diffraction data, radicals in the solid 2 form exchange-coupled chain have short contacts between nitroxide groups (NO....ON 3.904 Å, Supplementary Materials, Figure S2b). An analysis of the experimental µ_eff_(T) dependence on the uniform chain model gives best-fit values of g = 1.99 ± 0.01 and −0.60 ± 0.01 cm^−1^.

## 3. Materials and Methods

### 3.1. General Procedures and Materials

**Scheme 1:**
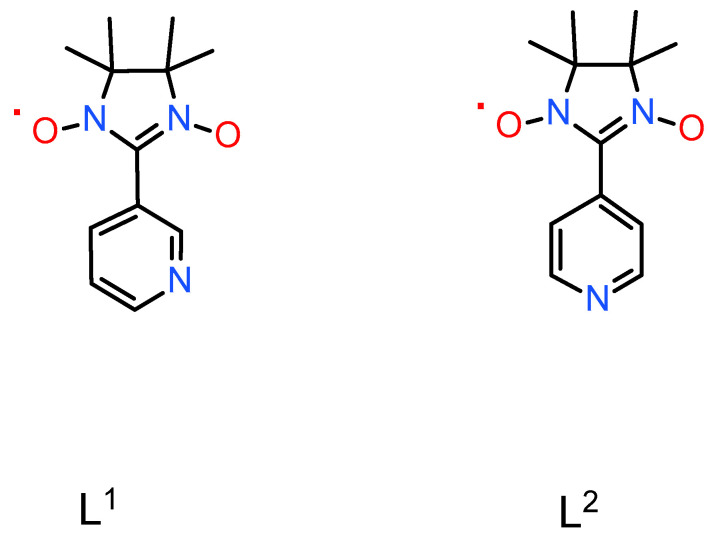
The ligands used in the investigation.

All reagents and solvents were of standard pure grade (Merck) and used without additional purification. Na_2_[RuNOCl_5_]·6H_2_O [44] and the nitroxide radicals [45,46] (Scheme 1) were prepared according to the techniques described earlier. The elemental analysis was conducted on a EuroVector EA3000 analyzer (EuroVector, Pavia, Italy). IR spectra in KBr pellets in the range of 400–4000 cm^−1^ were recorded on the spectrometer FCM 2201 “Infraspec”. 

### 3.2. Synthesis of Na[RuNOCl_4_L^1^]

Exact weights of Na_2_[RuNOCl_5_]·6H_2_O (46 mg, 0.1 mmol) and the ligand L^1^ (47 mg, 0.2 mmol) were mixed in 4 mL of acetonitrile, after that the mixture was stirred in a closely capped vial at 85 °C for 2 h. After cooling, the solution was filtered from NaCl and the blue filtrate was evaporated until dry at a reduced pressure. The final residue was dissolved in 1 mL of CH_2_Cl_2_, 4 mL of benzene was added, and the resulting suspension was stirred in a closely capped vial for 1 h at 75 °C. After cooling, the product precipitate was filtered, washed with benzene and diethyl ether, and air-dried. The final yield was 45 mg (80%). The IR spectra in cm^−1^ were: 2994(w), 2924(w), 2852(w), 1872(v.s.), 1452(m), 1402(m), 1372(s), 1324(m), 1215(m), 1177(m), 1135(m), 1068(m), 801(s), and 685(v).

NaRuCl_4_C_12_N_4_O_3_H_16_ found/calcd % were: N—10.3/10.6, C—27.1/27.2, and H—2.9/3.0.

Single crystals for X-ray analysis were prepared by the slow diffusion of diethyl ether in the acetonitrile solution. The different crystal phases for Na[RuNOCl_4_L^1^] were identified after diffusion in different conditions. Phase (**1**), prepared at 25 °C, is described earlier [19] and was stable in the absence of solvent. Phase (**2**), prepared at 5 °C, corresponds to the formula Na[RuNOCl_4_L^1^]·CH_3_CN and quickly loses solvent. For the magnetic measurements the stable phase (**1**) was used.

### 3.3. Synthesis of Na[RuNOCl_4_L^2^]·H_2_O

The complex was prepared using the same synthetic pathway as for the ligand L^1^. The final yield of blue-green precipitate was 40 mg (70%). IR spectra in sm^−1^ were: 3580(b.m.), 2994(w), 2924(w), 2852(w), 1868(v.s.), 1616(s), 1451(m), 1404(m), 1373(s), 1323(m), 1224(m), 1171(m), 1136(m), 1063(m), 1028(m), 832(s), and 689(v).

NaRuCl_4_C_12_N_4_O_4_H_18_ found/calcd % were: N—10.6/10.2, C—26.9/26.3, and H—3.6/3.3.

Single crystals for X-ray analysis were prepared by the slow diffusion of diethyl ether in the acetonitrile solution. Only one crystal phase Na[RuNOCl_4_L^2^]·H_2_O (**3**) was formed at temperatures of 25 and 5 °C and was used for the magnetic measurements.

### 3.4. Physical methods

The magnetic susceptibility of the polycrystalline samples was measured with a Quantum Design MPMSXL SQUID magnetometer in the temperature range 2–300 K with a magnetic field of up to 5 kOe. Diamagnetic corrections were made using the Pascal constants. The effective magnetic moment was calculated as μ_eff_(T) = [(3k/N_A_μ_B_^2^)χT]^1/2^ ≈ (8χT)^1/2^. The analysis of the experimental magnetic data was performed using the PHI program [47].

Mass spectrometric data were obtained on an Agilent liquid chromatograph-mass spectrometer system (LC-MS) 6130 Quadrupole MS, 1260 infinity LC. The analyses were performed in an *m/z* range from 200 to 2500 mass units, in SCAN mode for positive and negative ions. Electrospray ionization (ESI) was used as an ion source. The following parameters were used: nitrogen as a drying gas, a temperature of 350 °C, a flow rate of 7 L min^−1^, a spraying gas (nitrogen) pressure of 60 psig, and a voltage on the capillary of 4000 V. The voltage on the fragmentator was established to be 100 V in all experiments. A 5 µL acetonitrile (HPLC grade) solution of the studied compound with a concentration of 10^−4^–10^−3^ M was injected into the mobile phase, consisting of acetonitrile (special purity grade), at a rate of 0.4 mL min^−1^, sprayed, and ionized. The mass spectra (MS) were interpreted by matching signals to the proposed ions, including a comparison of the calculated and experimental isotopic peak distributions.

Cyclic voltammetry was carried out on an Elins P-20X8 voltammetry analyzer using a three-electrode scheme with a glassy carbon (GC) working electrode, a Pt auxiliary electrode, and an Ag/AgCl/3.5 M KCl reference electrode. Investigations were carried out for 2·10^−3^ M solutions of (**2**) and (**3**) with 0.1 M Bu_4_NBF_4_ in acetonitrile. Tetracyanoquinodimethane (TCNQ) was used as the internal standard.

The Hirshfeld surfaces were calculated using Crystal Explorer [48]. This program allows the normalized contact distance d_norm_ to be mapped onto the generated Hirshfeld surface. It is customary to map d_norm_ using a red–white–blue scheme, where red denotes close intermolecular contacts (negative d_norm_), blue denotes longer contacts (positive d_norm_), and white denotes intermolecular contacts equal to the van der Waals radii of atoms in contact (d_norm_ = 0). It is possible to obtain two-dimensional plots (fingerprint plots) from the surfaces mapped with d_norm_ values. Derived from the Hirshfeld surface, these 2D-fingerprint plots provide a visual summary of the frequency of each combination of d_e_ (radius of external atom) and d_i_ (radius of internal atom) across the surface of a molecule, so they not only indicate which intermolecular interactions are present, but also the relative area of the surface corresponding to each kind of interaction. Points on the plot with no contribution to the surface are left uncolored, and points with a contribution to the surface are colored blue for a small contribution through green to red for points with the largest contribution.

Single crystal X-ray diffraction data were collected by a Bruker Apex Duo diffractometer with CCDs using graphite-monochromated MoKα radiation (λ = 0.71073 Å) via 0.5° ω- and φ-scan techniques. An experimental data reduction was performed using the APEX2 suite. The structures were solved by SHELXT and refined by the full-matrix least-squares technique SHELXL [49]. Atomic displacement parameters of the non-H atoms were refined using anisotropic approximation. The hydrogen atoms of organic ligands were located geometrically and refined using the riding model. CCDC 2279338–2279339 contains the supplementary crystallographic data. The crystal data and structure refinement for the (**2**) and (**3**) are given in Table 4. These data can be obtained free of charge via http://www.ccdc.cam.ac.uk/conts/retrieving.html (accessed on 5 July 2023), or by e-mail.

## 4. Conclusions

The relatively simple synthetic approach allows us to combine the photochemically active ruthenium nitrosyl group with the magnetic nitronyl nitroxides in one coordination anion [RuNOCl_4_L]^−^. The prepared compounds are the first examples of the combination of inorganic (RuNO) and organic (nitroxides) NO groups in one coordination sphere. The crystal structure of sodium salts depends on the structure of nitroxide radicals, but the main features can be generalized. All the investigated crystal structures have 1D or 2D topology, due to the additional interactions between cations (Na^+^) and chloride ligands or the oxygen atoms of coordinated nitroxide ligands. Surprisingly the oligomeric fragments (n= 2, 3) with bridging sodium cations were also detected in acetonitrile solutions of the investigated complexes. The electrochemical behavior of the synthesized complexes demonstrates two quasi-reversible processes of the oxidation or reduction of nitroxide ligands and the irreversible oxidation of the (RuNO)^3+^ fragment. The irreversibility of (RuNO)^3+^ oxidation was well-established earlier, while the quasi-reversible redox processes of nitroxide ligands significantly differ from those of pure ligands. The magnetic properties of solid phases indicate that antiferromagnetic coupling at low temperatures goes through the exchange channels with short contacts between the paramagnetic centers of nitroxide ligands. Since the coordination fragment [RuNOCl_4_L]^−^ has an anionic nature, it could be combined further with different paramagnetic cations to prepare magnetic materials with different paramagnetic centers. Taking into account the potential photochemical activity of the RuNO group, these materials can demonstrate the photoswitching of magnetic properties.

## Figures and Tables

**Table 1:** Selected parameters in the structure of (**1**)–(**3**).

Parameter	NaRuNOCl_4_L ^1^ (1) [19]	NaRuNOCl_4_L ^1^ (2)	NaRuNOCl_4_L ^2^ (3)
d (Ru-Cl), Å	2.349(1)–2.398(1)	2.345(1)–2.382(1)	2.347(1)–2.400(1)
d (Ru-N_L_), Å	2.102(3)	2.111(2)–2.118(2)	2.110(4)
d (Ru-NO), Å	1.726(3)	1.736(3)–1.752(3)	1.799(4)
d (RuN-O), Å	1.133(4)	1.110(3)–1.124(3)	1.011(5)
∠Ru-N-O, °	178.1(3)	173.6(3)–179.0(3)	177.8(4)
d (N-O(L)), Å	1.275(3)–1.303(3)	1.273(3)–1.296(3)	1.260(5)–1.272(5)
∠NN-Py *, °	49.5(4)	41.0(4), 48.2(4)	33.5(2)

* An interplane angle between the pyridine ring and C_1_N_2_O_2_ plane of the nitroxide fragment.

**Table 2:** Main assigned forms in the mass-spectra (negative ions) of (**2**) and (**3**).

Complex Forms	(2)	(3)
	*m/z*	
RuNOCl_4_	274	274
RuNOCl_4_L^−^ (A)	508	508
(A)-O	492	492
NaRuNOCl_4_(RuNOCl_4_L) (B)	805	805
Na(RuNOCl_4_L)_2_ (C)	1039	1039
(C)-O	1023	1023
(C) + CH_3_CN	-	1080
Na_2_(RuNOCl_4_L)_3_ (D)	1568	1568
Na_3_(RuNOCl_4_L)_4_ (E)	2068	-

**Table 3:** Electrochemical characteristics of (**2**) and (**3**) and the corresponding assigned processes.

Scan Rate, V/s	E_pc,_ V		E_pa_, V		
	N-O·→ N-O^−^	N^+^ = O → N-O·	N-O^−^ → N-O·	N-O → N^+^ = O·	(Ru^2+^-NO^+^) →(Ru^3+^-NO^+^)
	Complex (**2**)				
0.1	−0.75	0.88	ND	1.26	1.36
0.25	−1.14	0.86	−0.65	0.97	1.28
0.5	−1.13	0.86	−0.64	0.98	1.31
	Complex (**3**)				
0.1	−0.73	0.93	ND	1.24	1.39
0.25	−1.09	0.92	−0.68	1.01	1.23
0.5	−1.10	0.90	−0.67	1.00	1.26

**Table 4:** Crystal data and structure refinement.

	(2)	(3)
Empirical formula	C_28_H_38_Cl_8_N_10_Na_2_O_6_Ru_2_	C_12_H_18_Cl_4_N_4_NaO_4_Ru
Formula weight	1142.40	548.16
Crystal system	triclinic	orthorhombic
Space group	P-1	P2_1_2_1_2_1_
a/Å	10.4158(2)	6.9913(2)
b/Å	10.8548(2)	15.1057(3)
c/Å	20.9285(4)	18.7215(5)
α/°	99.831(1)	90
β/°	94.477(1)	90
γ/°	109.036(1)	90
Volume/Å^3^	2181.12(7)	1977.15(9)
Z	2	4
ρ_calc_g/cm^3^	1.739	1.842
μ/mm^−1^	1.254	1.381
F(000)	1140.0	1092.0
2Θ range for data collection/°	4.714 to 66.502	3.464 to 66.392
Index ranges	−16 ≤ h ≤ 16, −16 ≤ k ≤ 16, −32 ≤ l ≤ 29	−10 ≤ h ≤ 10, −23 ≤ k ≤ 22, −28 ≤ l ≤ 28
Reflections collected	43,674	26,074
Independent reflections	16,301 [R_int_ = 0.0452, R_sigma_ = 0.0718]	7563 [Rint = 0.0388, Rsigma = 0.0426]
Data/restraints/parameters	16,301/0/515	7563/4/245
Goodness-of-fit on F^2^	0.861	1.058
Final R indexes [I ≥ 2σ (I)]	R_1_ = 0.0460, wR_2_ = 0.1217	R_1_ = 0.0400, wR_2_ = 0.0838
Final R indexes [all data]	R_1_ = 0.0835, wR_2_ = 0.1466	R_1_ = 0.0486, wR_2_ = 0.0875

## Data Availability

The data presented in this study are available on request from the corresponding author.

## Supplementary Materials

The following supporting information can be downloaded at: https://www.mdpi.com/article/10.3390/ijms241713371/s1.
