# Supplementary material for: New Ruthenium Nitrosyl Complexes Combining Potentially Photoactive Nitrosyl Group with the Magnetic Nitroxide Radicals as Ligands
Source: Int J Mol Sci. 2023 Aug 29;24(17):13371. doi: 10.3390/ijms241713371 (PMC10488014; doi:10.3390/ijms241713371)

checkCIF/PLATON report

Structure factors have been supplied for datablock(s) kru519

THIS REPORT IS FOR GUIDANCE ONLY. IF USED AS PART OF A REVIEW PROCEDURE FOR PUBLICATION, IT SHOULD NOT REPLACE THE EXPERTISE OF AN EXPERIENCED CRYSTALLOGRAPHIC REFEREE.

No syntax errors found. CIF dictionary Interpreting this report

Datablock: kru519

Bond precision:	C-C = 0.0046 A		Wavelength=0.71073
Cell:	a=10.4158(2)	b=10.8548(2)	c=20.9285(4)
	alpha=99.831(1)	beta=94.477(1)	gamma=109.036(1)
Temperature:	173 K		

	Calculated	Reported
Volume	2181.12(7)	2181.12(7)
Space group	P -1	P -1
Hall group	-P 1	-P 1
Moiety formula	C28 H38 Cl8 N10 Na2 O6 Ru2	C28 H38 Cl8 N10 Na2 O6 Ru2
Sum formula	C28 H38 Cl8 N10 Na2 O6 Ru2	C28 H38 Cl8 N10 Na2 O6 Ru2
Mr	1142.41	1142.40
Dx,g cm-3	1.740	1.739
Z	2	2
Mu (mm-1)	1.254	1.254
F000	1140.0	1140.0
F000'	1137.73	
h,k,lmax	16,16,32	16,16,32
Nref	16767	16301
Tmin,Tmax	0.900,0.963	0.648,0.747
Tmin'	0.778	

Correction method= # Reported T Limits: Tmin=0.648 Tmax=0.747
AbsCorr = MULTI-SCAN

Data completeness= 0.972 Theta(max)= 33.251

R(reflections)= 0.0460(11225) wR2(reflections)= 0.1466(16301)

S = 0.861 Npar= 515

The following ALERTS were generated. Each ALERT has the format
test-name_ALERT_alert-type_alert-level.
Click on the hyperlinks for more details of the test.

Alert level C

```
PLAT220_ALERT_2_C NonSolvent   Resd 1  C   Ueq(max)/Ueq(min) Range        4.9 Ratio
PLAT220_ALERT_2_C NonSolvent   Resd 1  N   Ueq(max)/Ueq(min) Range        4.7 Ratio
PLAT241_ALERT_2_C High  'MainMol' Ueq as Compared to Neighbors of        N1A Check
PLAT906_ALERT_3_C Large K Value in the Analysis of Variance ......      2.080 Check
PLAT910_ALERT_3_C Missing # of FCF Reflection(s) Below Theta(Min).          6 Note
PLAT911_ALERT_3_C Missing FCF Refl Between Thmin & STh/L=    0.600          2 Report
PLAT971_ALERT_2_C Check Calcd Resid. Dens.  0.81Ang From N2N             1.61 eA-3
```

Alert level G

```
PLAT004_ALERT_5_G Polymeric Structure Found with Maximum Dimension          2 Info
PLAT154_ALERT_1_G The s.u.'s on the Cell Angles are Equal ..(Note)      0.001 Degree
PLAT171_ALERT_4_G The CIF-Embedded .res File Contains EADP Records          1 Report
PLAT232_ALERT_2_G Hirshfeld Test Diff (M-X)  Ru1      --N1N      .        7.4 s.u.
PLAT232_ALERT_2_G Hirshfeld Test Diff (M-X)  Ru2      --Cl5      .        6.0 s.u.
PLAT232_ALERT_2_G Hirshfeld Test Diff (M-X)  Ru2      --Cl6      .        6.8 s.u.
PLAT232_ALERT_2_G Hirshfeld Test Diff (M-X)  Ru2      --Cl7      .        5.5 s.u.
PLAT232_ALERT_2_G Hirshfeld Test Diff (M-X)  Ru2      --N2N      .        5.6 s.u.
PLAT300_ALERT_4_G Atom Site Occupancy of Na2       Constrained at         0.5 Check
PLAT301_ALERT_3_G Main Residue  Disorder ..............(Resd  1  )        1% Note
PLAT431_ALERT_2_G Short Inter HL..A Contact  Cl6      ..O2N      .       3.16 Ang.
                                                1-x,-y,1-z  =      2_656 Check
PLAT720_ALERT_4_G Number of Unusual/Non-Standard Labels .........           6 Note
PLAT779_ALERT_4_G Suspect or Irrelevant (Bond) Angle(s) in CIF ...      25.80 Deg.
              NA2  -O43  -NA2     2_655   1_555   1_555 ......     #  189 Check
PLAT779_ALERT_4_G Suspect or Irrelevant (Bond) Angle(s) in CIF ...      23.72 Deg.
              NA2  -N1A  -NA2     2_655   1_555   1_555 ......     #  271 Check
PLAT912_ALERT_4_G Missing # of FCF Reflections Above STh/L=  0.600        460 Note
PLAT933_ALERT_2_G Number of HKL-OMIT Records in Embedded .res File          4 Note
PLAT941_ALERT_3_G Average HKL Measurement Multiplicity ..........         2.7 Low
PLAT978_ALERT_2_G Number C-C Bonds with Positive Residual Density.          2 Info
```

0 **ALERT level A** = Most likely a serious problem - resolve or explain
0 **ALERT level B** = A potentially serious problem, consider carefully
7 **ALERT level C** = Check. Ensure it is not caused by an omission or oversight
18 **ALERT level G** = General information/check it is not something unexpected

1 ALERT type 1 CIF construction/syntax error, inconsistent or missing data
12 ALERT type 2 Indicator that the structure model may be wrong or deficient
5 ALERT type 3 Indicator that the structure quality may be low
6 ALERT type 4 Improvement, methodology, query or suggestion
1 ALERT type 5 Informative message, check

It is advisable to attempt to resolve as many as possible of the alerts in all categories. Often the minor alerts point to easily fixed oversights, errors and omissions in your CIF or refinement strategy, so attention to these fine details can be worthwhile. In order to resolve some of the more serious problems it may be necessary to carry out additional measurements or structure refinements. However, the purpose of your study may justify the reported deviations and the more serious of these should normally be commented upon in the discussion or experimental section of a paper or in the "special_details" fields of the CIF. checkCIF was carefully designed to identify outliers and unusual parameters, but every test has its limitations and alerts that are not important in a particular case may appear. Conversely, the absence of alerts does not guarantee there are no aspects of the results needing attention. It is up to the individual to critically assess their own results and, if necessary, seek expert advice.

Publication of your CIF in IUCr journals

A basic structural check has been run on your CIF. These basic checks will be run on all CIFs submitted for publication in IUCr journals (*Acta Crystallographica*, *Journal of Applied Crystallography*, *Journal of Synchrotron Radiation*); however, if you intend to submit to *Acta Crystallographica Section C* or *E* or *IUCrData*, you should make sure that full publication checks are run on the final version of your CIF prior to submission.

Publication of your CIF in other journals

Please refer to the *Notes for Authors* of the relevant journal for any special instructions relating to CIF submission.

PLATON version of 10/05/2023; check.def file version of 10/05/2023

Datablock kru519 - ellipsoid plot

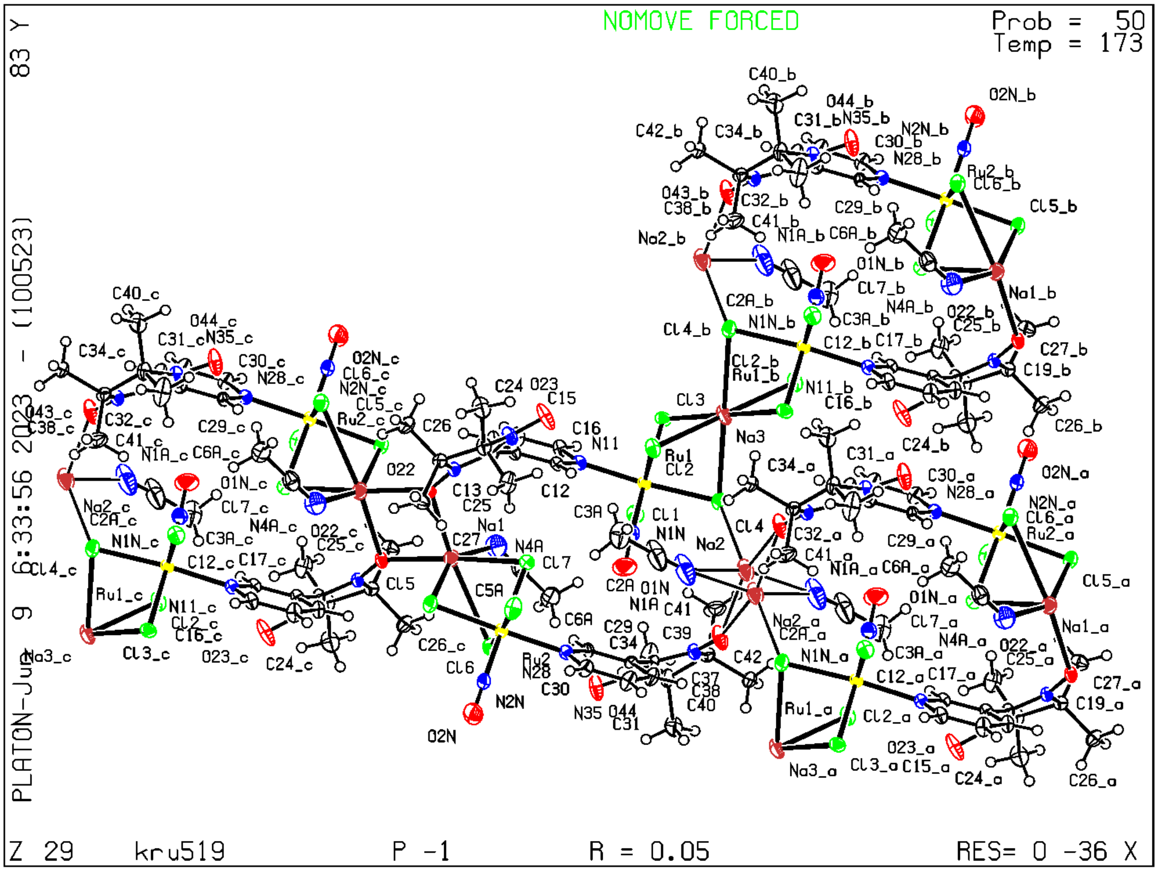

Supplement: Supplementary file 1 [file ijms-24-13371-s001.zip › checkcif1.pdf]
