# Supplementary material for: New Ruthenium Nitrosyl Complexes Combining Potentially Photoactive Nitrosyl Group with the Magnetic Nitroxide Radicals as Ligands
Source: Int J Mol Sci. 2023 Aug 29;24(17):13371. doi: 10.3390/ijms241713371 (PMC10488014; doi:10.3390/ijms241713371)

checkCIF/PLATON report

Structure factors have been supplied for datablock(s) pb002

THIS REPORT IS FOR GUIDANCE ONLY. IF USED AS PART OF A REVIEW PROCEDURE FOR PUBLICATION, IT SHOULD NOT REPLACE THE EXPERTISE OF AN EXPERIENCED CRYSTALLOGRAPHIC REFEREE.

No syntax errors found. CIF dictionary Interpreting this report

Datablock: pb002

```
Bond precision:   C-C = 0.0062 A          Wavelength=0.71073

Cell:               a=6.9913(2)      b=15.1057(3)      c=18.7215(5)
                    alpha=90         beta=90           gamma=90
Temperature:        150 K

                 Calculated                   Reported
Volume           1977.15(9)                   1977.15(9)
Space group      P 21 21 21                   P 21 21 21
Hall group       P 2ac 2ab                    P 2ac 2ab
Moiety formula   C12 H18 Cl4 N4 Na O4 Ru      C12 H18 Cl4 N4 Na O4 Ru
Sum formula      C12 H18 Cl4 N4 Na O4 Ru      C12 H18 Cl4 N4 Na O4 Ru
Mr               548.16                       548.16
Dx,g cm-3        1.842                        1.842
Z                4                            4
Mu (mm-1)        1.381                        1.381
F000             1092.0                       1092.0
F000'            1089.75
h,k,lmax         10,23,28                     10,23,28
Nref             7575[ 4254]                  7563
Tmin,Tmax        0.824,0.933                  0.598,0.747
Tmin'            0.824

Correction method= # Reported T Limits: Tmin=0.598 Tmax=0.747
AbsCorr = MULTI-SCAN

Data completeness= 1.78/1.00       Theta(max)= 33.196

R(reflections)= 0.0399( 6751)                          wR2(reflections)=
                                                       0.0873( 7563)
S = 1.060                   Npar= 245
```

The following ALERTS were generated. Each ALERT has the format
test-name_ALERT_alert-type_alert-level.
Click on the hyperlinks for more details of the test.

Alert level C

```
PLAT230_ALERT_2_C Hirshfeld Test Diff for    O2N      --N1N      .        5.7 s.u.
PLAT976_ALERT_2_C Check Calcd Resid. Dens.  0.47Ang From O1W     .      -0.53 eA-3
```

Alert level G

```
PLAT002_ALERT_2_G Number of Distance or Angle Restraints on AtSite          3 Note
PLAT004_ALERT_5_G Polymeric Structure Found with Maximum Dimension          1 Info
PLAT172_ALERT_4_G The CIF-Embedded .res File Contains DFIX Records          2 Report
PLAT232_ALERT_2_G Hirshfeld Test Diff (M-X)  Ru1      --Cl1      .        7.0 s.u.
PLAT232_ALERT_2_G Hirshfeld Test Diff (M-X)  Ru1      --Cl3      .       14.2 s.u.
PLAT232_ALERT_2_G Hirshfeld Test Diff (M-X)  Ru1      --N1N      .       16.6 s.u.
PLAT720_ALERT_4_G Number of Unusual/Non-Standard Labels ..........          2 Note
PLAT860_ALERT_3_G Number of Least-Squares Restraints .............          2 Note
PLAT912_ALERT_4_G Missing # of FCF Reflections Above STh/L=  0.600          6 Note
PLAT978_ALERT_2_G Number C-C Bonds with Positive Residual Density.          2 Info
```

0 **ALERT level A** = Most likely a serious problem - resolve or explain
0 **ALERT level B** = A potentially serious problem, consider carefully
2 **ALERT level C** = Check. Ensure it is not caused by an omission or oversight
10 **ALERT level G** = General information/check it is not something unexpected

0 ALERT type 1 CIF construction/syntax error, inconsistent or missing data
7 ALERT type 2 Indicator that the structure model may be wrong or deficient
1 ALERT type 3 Indicator that the structure quality may be low
3 ALERT type 4 Improvement, methodology, query or suggestion
1 ALERT type 5 Informative message, check

It is advisable to attempt to resolve as many as possible of the alerts in all categories. Often the minor alerts point to easily fixed oversights, errors and omissions in your CIF or refinement strategy, so attention to these fine details can be worthwhile. In order to resolve some of the more serious problems it may be necessary to carry out additional measurements or structure refinements. However, the purpose of your study may justify the reported deviations and the more serious of these should normally be commented upon in the discussion or experimental section of a paper or in the "special_details" fields of the CIF. checkCIF was carefully designed to identify outliers and unusual parameters, but every test has its limitations and alerts that are not important in a particular case may appear. Conversely, the absence of alerts does not guarantee there are no aspects of the results needing attention. It is up to the individual to critically assess their own results and, if necessary, seek expert advice.

Publication of your CIF in IUCr journals

A basic structural check has been run on your CIF. These basic checks will be run on all CIFs submitted for publication in IUCr journals (*Acta Crystallographica*, *Journal of Applied Crystallography*, *Journal of Synchrotron Radiation*); however, if you intend to submit to *Acta Crystallographica Section C* or *E* or *IUCrData*, you should make sure that full publication checks are run on the final version of your CIF prior to submission.

Publication of your CIF in other journals

Please refer to the *Notes for Authors* of the relevant journal for any special instructions relating to CIF submission.

PLATON version of 10/05/2023; check.def file version of 10/05/2023

Datablock pb002 - ellipsoid plot

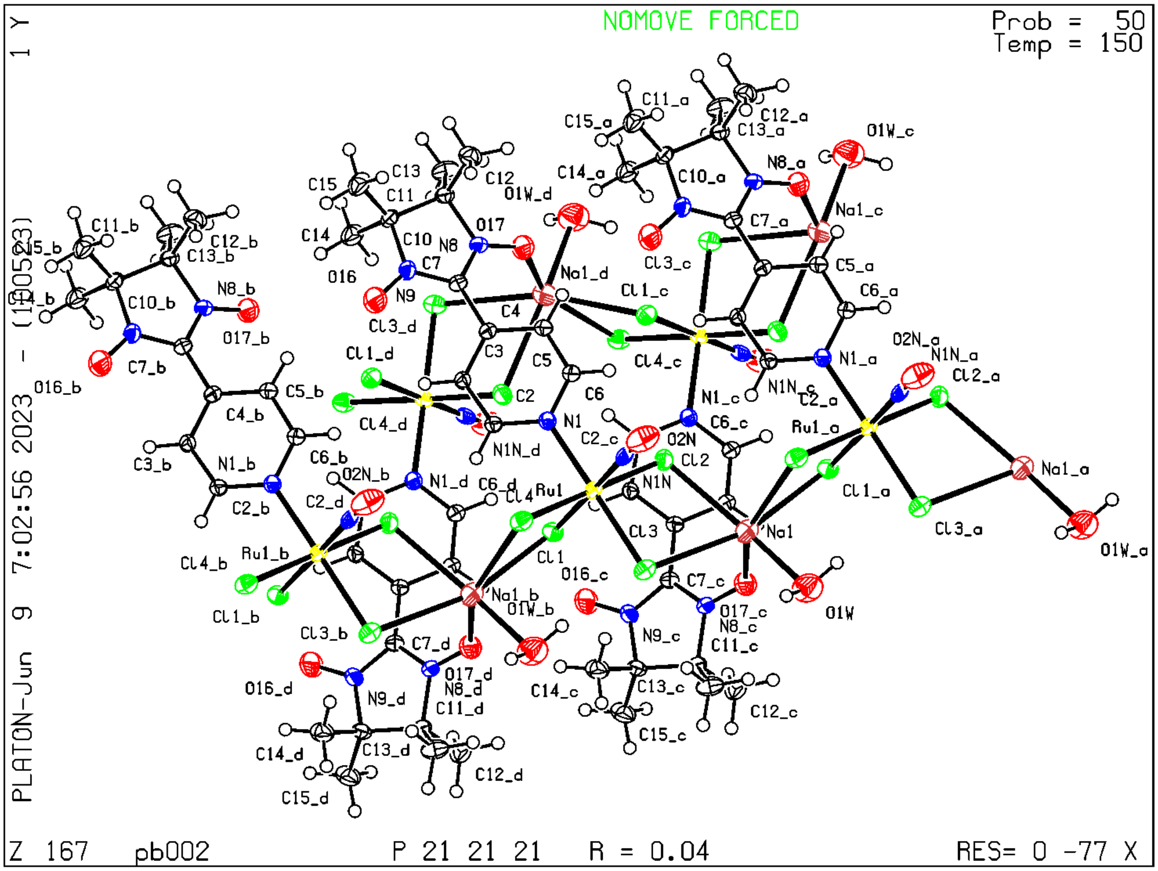

Supplement: Supplementary file 1 [file ijms-24-13371-s001.zip › checkcif2.pdf]
