# Supplementary material for: New Ruthenium Nitrosyl Complexes Combining Potentially Photoactive Nitrosyl Group with the Magnetic Nitroxide Radicals as Ligands
Source: Int J Mol Sci. 2023 Aug 29;24(17):13371. doi: 10.3390/ijms241713371 (PMC10488014; doi:10.3390/ijms241713371)
Supplement: Supplementary file 1 [file ijms-24-13371-s001.zip › SI.pdf]

Supplementary information to the article

«NEW RUTHENIUM NITROSYL COMPLEXES COMBINING POTENTIALLY PHOTOACTIVE NITROSYL GROUP WITH THE MAGNETIC NITROXIDE RADICALS AS LIGANDS.»

Gennadiy Kostin[1*], Ruslan Kozlov[2], Artem Bogomyakov[3], Svyatoslav Tolstikov[3], Dmitriy Sheven[1], Sergey Korenev[1]

1. ESI – MS data for the acetonitrile solutions of Na[RuNOCL4L]

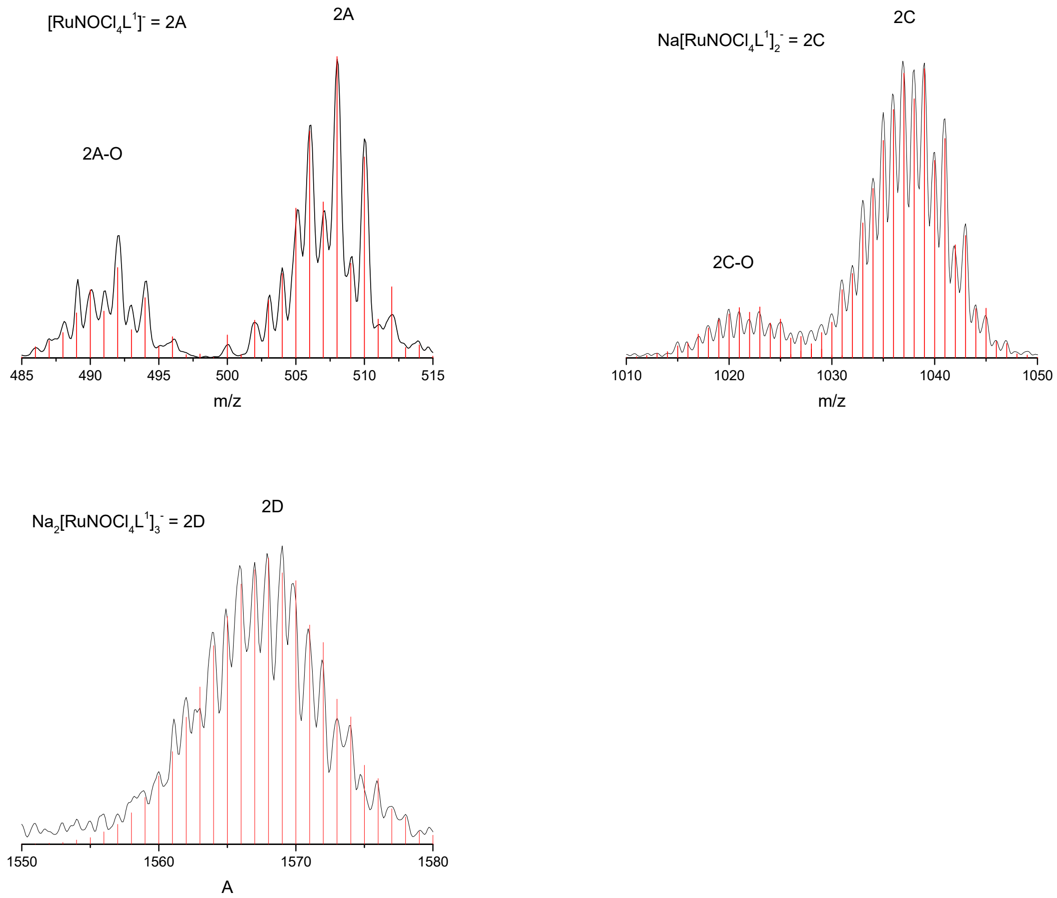

Fig. S1.a. Fragments of negative ions mass spectrum of Na[RuNOCl4L[1]] corresponding to main forms – experimental (lines) and theoretical isotopic distribution pattern.

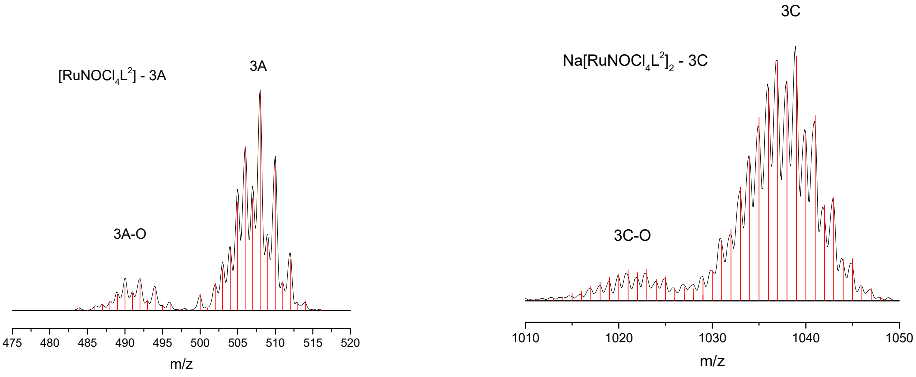

Fig. S1.b. Fragments of negative ions mass spectrum of Na[RuNOCl4L[2]] corresponding to main forms – experimental (lines) and theoretical isotopic distribution pattern.

2. The pictures of complex crystal structure with distances between paramagnetic centers.

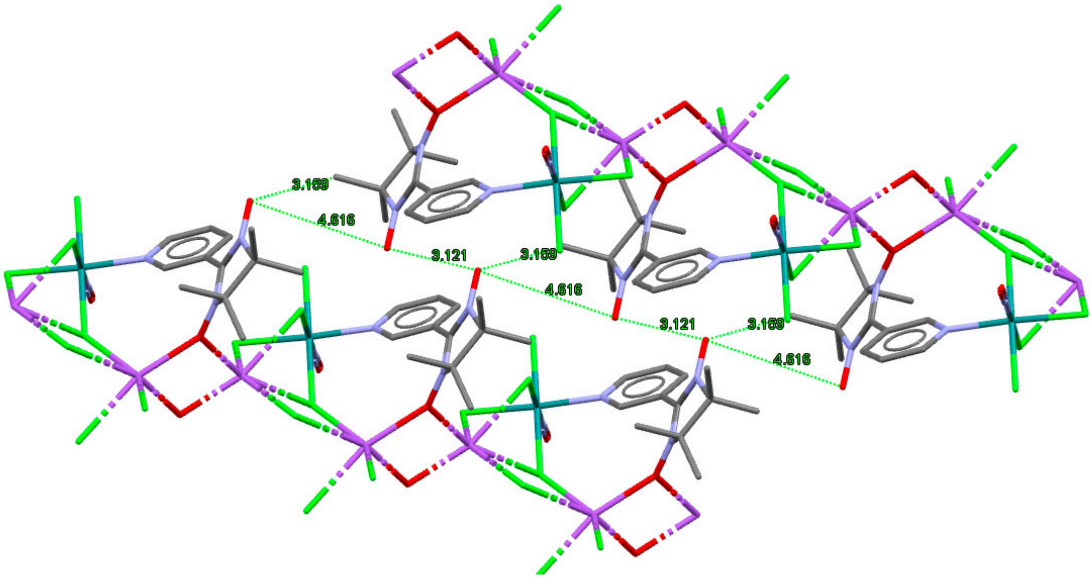

Fig. S.2.a. The crystal structure of $Na[RuNOCl_4L^1]$

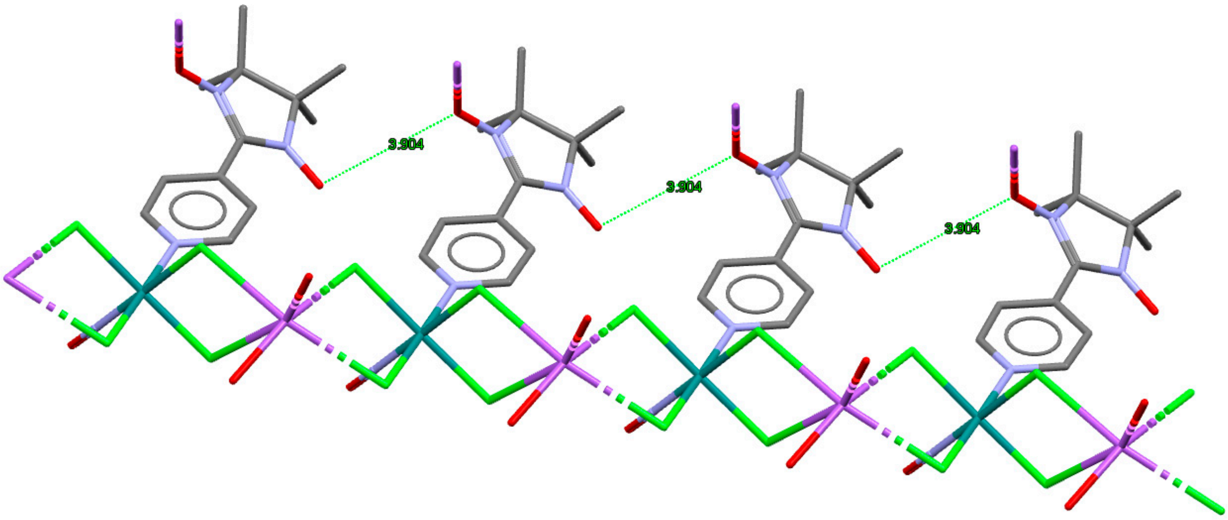

Fig. S.2.b. The crystal structure of $Na[RuNOCl_4L^2]$

3. Hirshfeld surfaces analysis for complexes **(2)** and **(3)**

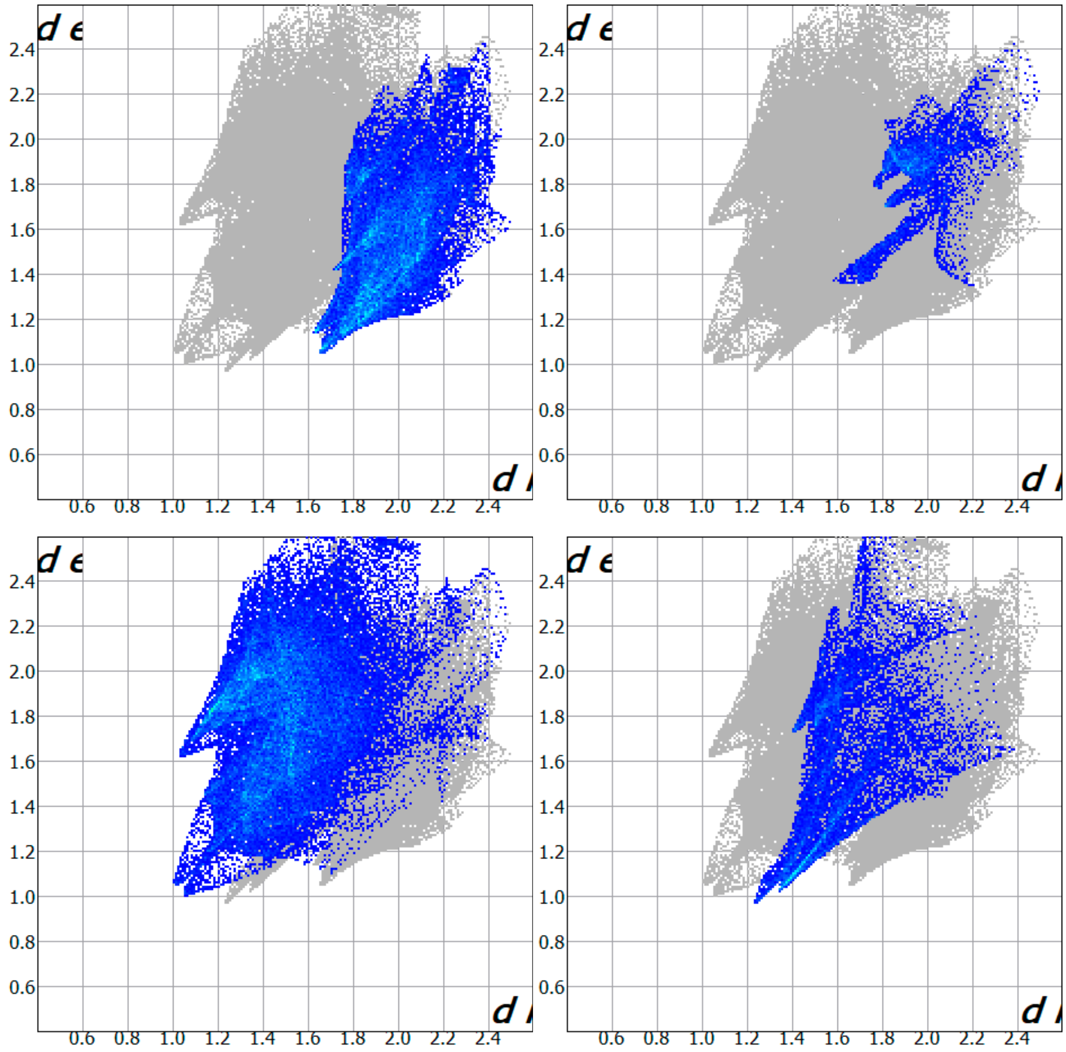

Figure S3.a. The 2D fingerprints patterns for the Hirshfeld surfaces **(2)** chlorine (upper left), carbon (upper right), hydrogen (low left) and oxygen (low right)

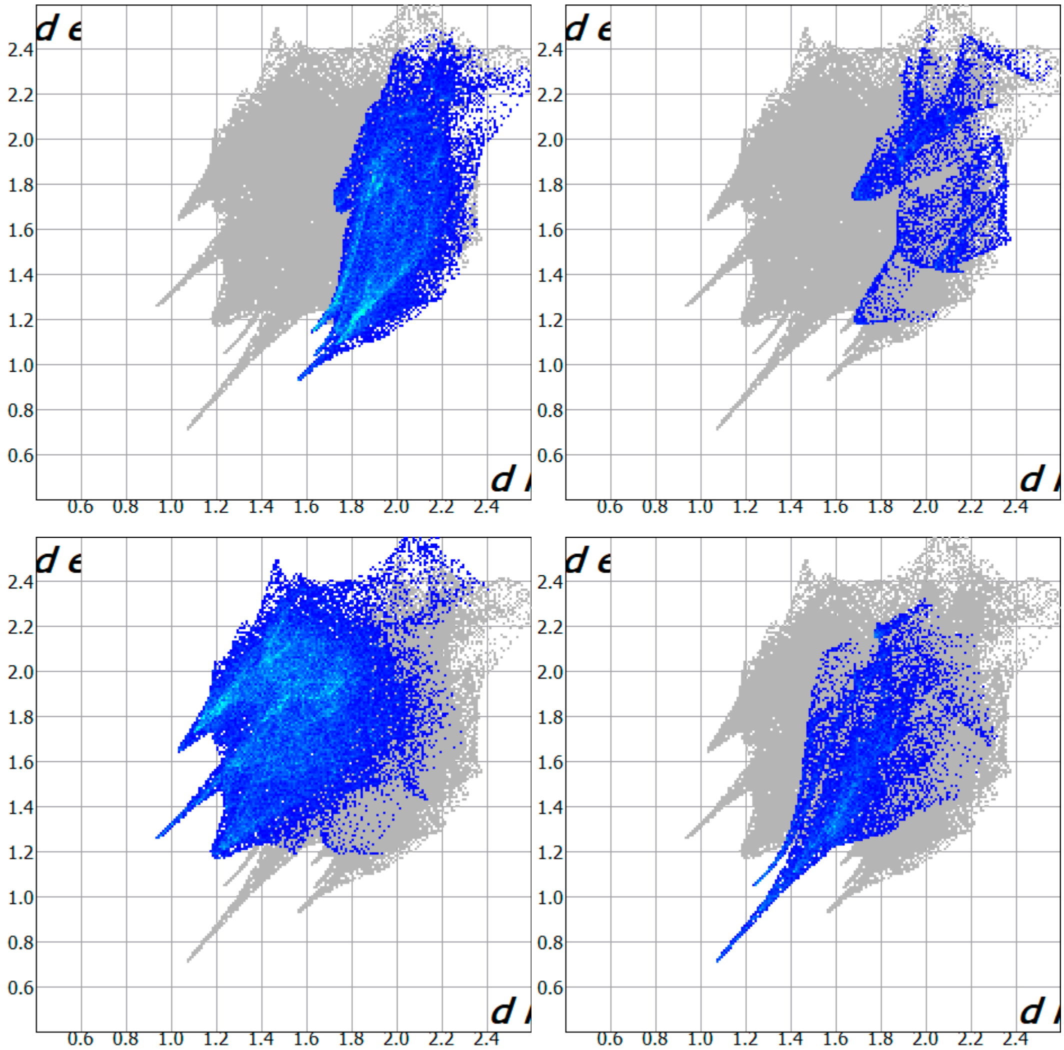

Figure S3.b. The 2D fingerprints patterns for the Hirshfeld surfaces **(3)** chlorine (upper left), carbon (upper right), hydrogen (low left) and oxygen (low right)
